# Supplementary figures and images for: Downregulation of OIP5-AS1 inhibits apoptosis in myocardial ischemia/reperfusion injury via modulating the MiR-145-5p/ROCK1 axis
Source: PLoS One. 2025 May 20;20(5):e0324909. doi: 10.1371/journal.pone.0324909 (PMC12091815; doi:10.1371/journal.pone.0324909)

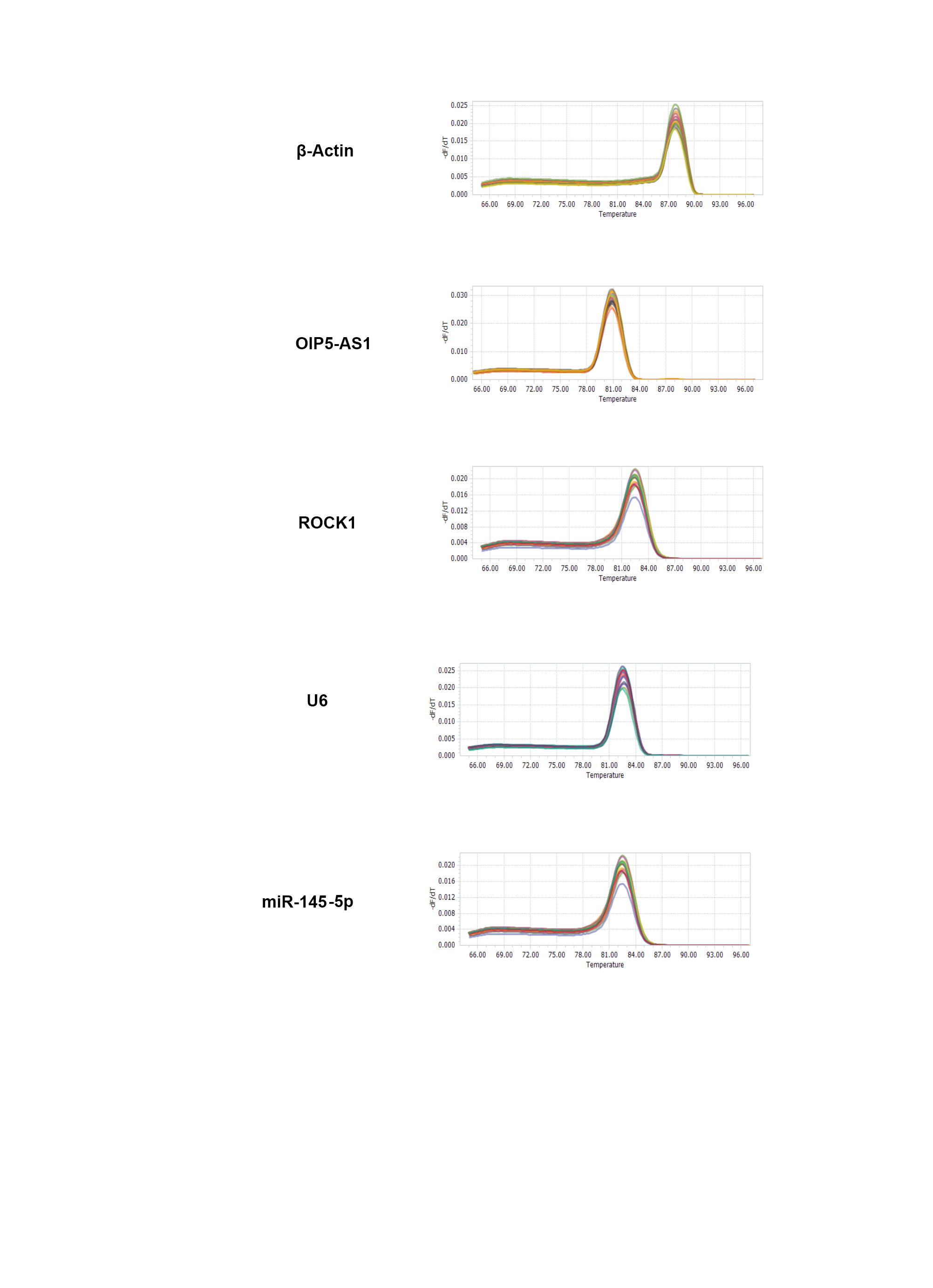

Supplement: S2 File — (TIF) [file pone.0324909.s002.tif]

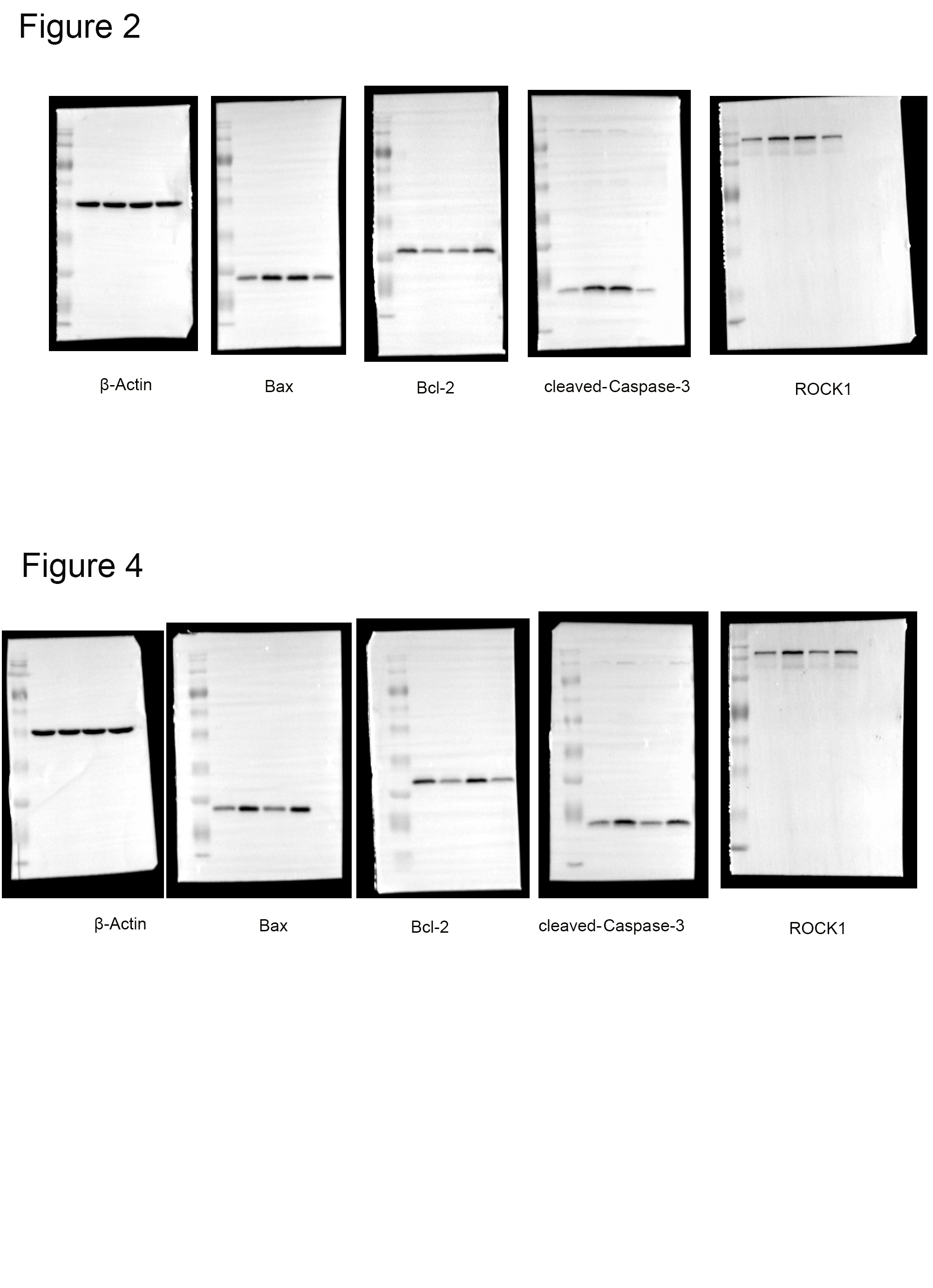

Supplement: S3 File — (TIF) [file pone.0324909.s003.tif]
