# Supplementary material for: Downregulation of OIP5-AS1 inhibits apoptosis in myocardial ischemia/reperfusion injury via modulating the MiR-145-5p/ROCK1 axis
Source: PLoS One. 2025 May 20;20(5):e0324909. doi: 10.1371/journal.pone.0324909 (PMC12091815; doi:10.1371/journal.pone.0324909)
Supplement: Attachment — Submitted filename: Response to Reviewers.docx [file pone.0324909.s005.docx]

Dear Editor:

Thank you very much for your letter and advice about our manuscript with the mumber of PONE-D-25-10451. According with reviewer’s comments, we revised the relevant parts, and these parts were highlighted in the manuscript. All of the questions were answered below.

**Journal Requirements:**

Please check the reviewers' comments. Ensure that all figures are appropriately cited and that the methods are described in sufficient detail for reproducibility. Include catalog IDs for all products used, as well as the sequences for the miR-145-5p mimic. Specify which method was used for the 2^-ΔΔCq calculation (Livak, Pfaffl, or another approach) and provide the corresponding citation. Clearly describe the procedure for adaptor ligation during miRNA-to-cDNA conversion. Define all abbreviations upon their first mention and thoroughly review the English grammar throughout the document. Include melting curves to confirm the specific amplification of all target regions, including miR-145-5p. Replace bar plots with box plots or violin plots, supplemented by point or jitter plots to display all biological replicates, particularly for expression analyses, as this provides a more transparent representation of the results.

We have reviewed and addressed all reviewers' comments point-by-point in this revision.We have included the melting curve analysis for target amplifications in the supplementary materials.We have verified that all figures are cited in sequential order and match the text descriptions, and we replaced all bar plots with box plots + superimposed jittered points. We defined all abbreviations at first use (e.g., ceRNA: competing endogenous RNA).

When submitting your revision, we need you to address these additional requirements.

1. Please ensure that your manuscript meets PLOS ONE's style requirements, including those for file naming.

Yes,We are sure that our manuscript meets PLOS ONE's style requirements.

2. To comply with PLOS ONE submissions requirements, in your Methods section, please provide additional information regarding the experiments involving animals and ensure you have included details on (1) methods of sacrifice, (2) methods of anesthesia and/or analgesia, and (3) efforts to alleviate suffering.

I would like to confirm that we have already incorporated the necessary information regarding the animal experiments in our Methods section to comply with the submission requirements of PLOS ONE. Specifically, we have detailed the following aspects: (1) methods of sacrifice. We have described the specific techniques used to euthanize the animals at the end of the experiments, ensuring that the methods were conducted in a manner that minimized pain and distress. At the conclusion of the experiments, animals were euthanized with an overdose of isoflurane administered for approximately 10 minutes, followed by euthanasia through exsanguination. (2) methods of anesthesia and/or analgesia. Animalss were anaesthetized with pentobarbital (50mg/kg, i.p.) and mechanically ventilated. (3) efforts to alleviate suffering. Efforts were made to minimize animal suffering and to use the minimum number of animals required to achieve statistical significance.

3. Thank you for stating the following financial disclosure:

“Natural Science Foundation of Shandong Province (grant no. ZR2022MH285)”

Please state what role the funders took in the study. If the funders had no role, please state: "The funders had no role in study design, data collection and analysis, decision to publish, or preparation of the manuscript."

If this statement is not correct you must amend it as needed.

Please include this amended Role of Funder statement in your cover letter; we will change the online submission form on your behalf.

We state that "The funders had no role in study design, data collection and analysis, decision to publish, or preparation of the manuscript."

4. Thank you for stating the following in the Funding Section of your manuscript:

“The authors gratefully acknowledge research support provided by Natural Science Foundation of Shandong Province (grant no. ZR2022MH285).”

We note that you have provided funding information that is currently declared in your Funding Statement. However, funding information should not appear in the Acknowledgments section or other areas of your manuscript. We will only publish funding information present in the Funding Statement section of the online submission form.

Please remove any funding-related text from the manuscript and let us know how you would like to update your Funding Statement. Currently, your Funding Statement reads as follows:

“Natural Science Foundation of Shandong Province (grant no. ZR2022MH285)”

Please include your amended statements within your cover letter; we will change the online submission form on your behalf.

We have removed funding-related text from the revised manuscript.

The authors gratefully acknowledge research support provided by Natural Science Foundation of Shandong Province (grant no. ZR2022MH285). The funders had no role in study design, data collection and analysis, decision to publish, or preparation of the manuscript.

5. When completing the data availability statement of the submission form, you indicated that you will make your data available on acceptance. We strongly recommend all authors decide on a data sharing plan before acceptance, as the process can be lengthy and hold up publication timelines. Please note that, though access restrictions are acceptable now, your entire data will need to be made freely accessible if your manuscript is accepted for publication. This policy applies to all data except where public deposition would breach compliance with the protocol approved by your research ethics board. If you are unable to adhere to our open data policy, please kindly revise your statement to explain your reasoning and we will seek the editor's input on an exemption. Please be assured that, once you have provided your new statement, the assessment of your exemption will not hold up the peer review process.

We fully understand the significance of having a clear data sharing plan in place and its impact on the publication process. After careful consideration, we have decided to adhere to your open data policy and are committed to making our entire dataset freely accessible upon acceptance of our manuscript, with the exception of any data that would breach compliance with our approved research ethics protocol.

We are currently in the process of preparing our data for public deposition and will ensure that all necessary steps are taken to meet the required standards. We have also uploaded the raw data in the supplementary materials.

If there are any specific requirements or guidelines that we should follow to facilitate this process, please do not hesitate to let us know. We are more than willing to comply and ensure a smooth transition towards open data sharing.

6. PLOS ONE now requires that authors provide the original uncropped and unadjusted images underlying all blot or gel results reported in a submission’s figures or Supporting Information files. This policy and the journal’s other requirements for blot/gel reporting and figure preparation are described in detail at https://journals.plos.org/plosone/s/figures#loc-blot-and-gel-reporting-requirements and https://journals.plos.org/plosone/s/figures#loc-preparing-figures-from-image-files. When you submit your revised manuscript, please ensure that your figures adhere fully to these guidelines and provide the original underlying images for all blot or gel data reported in your submission. See the following link for instructions on providing the original image data: https://journals.plos.org/plosone/s/figures#loc-original-images-for-blots-and-gels.

In your cover letter, please note whether your blot/gel image data are in Supporting Information or posted at a public data repository, provide the repository URL if relevant, and provide specific details as to which raw blot/gel images, if any, are not available. Email us at plosone@plos.org if you have any questions

We have carefully reviewed the guidelines provided in the links and have ensured that our figures fully comply with the PLOS ONE requirements for blot/gel reporting and figure preparation. The original uncropped and unadjusted images underlying all blot or gel results reported in our submission have been submitted.

We have already uploaded the original blot and gel image data as part of the Supporting Information.

6. Your ethics statement should only appear in the Methods section of your manuscript. If your ethics statement is written in any section besides the Methods, please delete it from any other section.

We have removed the ethics statement that appeared elsewhere in the manuscript as requested.

7. Please include captions for your Supporting Information files at the end of your manuscript, and update any in-text citations to match accordingly.

We have included captions for all Supporting Information files at the end of our manuscript and have updated the in-text citations to match accordingly.

**Reviewer #1:**

Yang and his colleagues observed that the expression of the long non-coding RNA (lncRNA) OIP5-AS1 and apoptosis were increased in myocardial ischemia/reperfusion (I/R) injury. Based on their previous study, they further confirmed that the expression of miR-145-5p was increased, while its target, ROCK1, was decreased. They then demonstrated that reducing OIP5-AS1 expression led to a decrease in apoptosis and experimentally validated the relationship between OIP5-AS1 and miR-145-5p.

The paper presents a very straightforward mechanism, and the experimental evidence supporting each claim is convincing.

However, to strengthen the authors' argument, I suggest including additional experiments to clarify the relationship between ROCK1 and OIP5-AS1:
1. Does overexpression of ROCK1 in the context of reduced OIP5-AS1 modulate the level of apoptosis?

We sincerely appreciate the reviewer’s insightful suggestion to further strengthen the mechanistic link between OIP5-AS1 and ROCK1 in regulating apoptosis. We overexpressed ROCK1 in OIP5-AS1-deficient cells. In Fig7, while OIP5-AS1 knockdown alone reduced apoptosis, concomitant ROCK1 overexpression reversed this effect.

2. How does apoptosis change when anti-miR-145-5p is introduced in a setting where OIP5-AS1 is downregulated?

In Fig6 of the manuscript, we investigated whether OIP5-AS1 regulates ischemia/reperfusion (I/R)-induced apoptosis through miR-145-5p. TUNEL staining results demonstrated that suppression of OIP5-AS1 significantly reduced cellular apoptosis, whereas treatment with a miR-145-5p inhibitor completely abolished this anti-apoptotic effect.

**Reviewer #2:**

Thank you for the opportunity to review your manuscript titled "Downregulation of OIP5-AS1 Inhibits Apoptosis in Myocardial Ischemia/Reperfusion Injury via Modulating the MiR-145-5p/ROCK1 Axis." Your study addresses an important and relevant area of research with potential implications for novel therapeutic strategies targeting myocardial ischemia/reperfusion injury. The exploration of OIP5-AS1's interaction with miR-145-5p and its downstream impact on ROCK1 is promising and could significantly contribute to the existing literature.

However, the methodology section needs substantial improvement. The descriptions provided are too simplified and lack critical details necessary for replication. Specifically, information regarding the exact concentrations of siRNAs, miRNA mimics, and inhibitors utilized in the experiments is missing. Additionally, you should justify the choice of Lipofectamine 2000 over the more RNA-specific reagent Lipofectamine RNAiMAX. Clarification about whether optimization experiments using "killer" siRNA were conducted would also strengthen your methodological transparency. Including control experiments with Lipofectamine alone would clarify if observed effects are due specifically to the treatments or influenced by reagent toxicity. Also, crucial details such as the quantity of RNA used in qPCR reactions, conditions for replicates (technical or biological), the acceptable Ct variation between replicates, and explicitly mentioning the normalization gene used (presumably snU6) must be clearly stated.

Thank you for your valuable advice. We acknowledge the need for a more detailed and transparent methodology section to ensure reproducibility and clarity.

We have added specific information regarding the use of siRNAs, miRNA mimics, and inhibitors in the manuscript. Lipofectamine 2000 was chosen for its proven efficacy, compatibility with our cell model, and alignment with prior research methodologies. This decision was further supported by optimization experiments and appropriate controls to ensure the reliability of our findings.

Optimization studies were performed using a cytotoxicity-inducing siRNA (a non-targeting siRNA with a known cytotoxic effect) to establish the optimal transfection conditions while minimizing cell toxicity. The final transfection conditions were selected based on achieving a knockdown efficiency exceeding 80% while maintaining cell viability above 90%.

For qPCR experiments, Acceptable Ct variation between replicates: ≤0.5. U6 was used as the endogenous control for normalization of miRNA expression levels. For mRNA quantification, Actin was used as the housekeeping gene.

In your results section, findings are presented clearly and accompanied by suitable statistical analyses. Nevertheless, minor typographical errors were identified—for instance, "Conrtol" should be corrected to "Control" in Figure 2. Moreover, please clarify whether control groups containing only Lipofectamine (without siRNAs or mimics) were included to exclude nonspecific reagent effects.

Regarding the typographical error in Figure , we acknowledge the mistake and confirm that "Conrtol" will be corrected to "Control" in the revised version of the manuscript. We will thoroughly proofread the entire document to ensure no similar errors remain. Thank you again for your attention to detail and constructive suggestions. And we confirm that control groups containing only Lipofectamine (without siRNAs or mimics) were indeed included in all experiments.

Your discussion section is somewhat superficial and would greatly benefit from a more thorough examination of the study's limitations. It is essential to explicitly address limitations, such as small sample sizes, potential off-target effects of transfection agents, and methodological constraints inherent to the model systems used. Additionally, clearly stating future research directions would significantly strengthen this section. For example, suggesting studies that could confirm your findings in larger or more clinically relevant contexts would substantially enhance the manuscript’s scientific and clinical significance.
Furthermore, you should provide clear steps or recommendations to explore the clinical applicability of your findings. For instance, how might the OIP5-AS1 and miR-145-5p/ROCK1 axis modulation translate into clinical interventions or therapeutic approaches?

We sincerely appreciate the reviewer’s constructive feedback. In response to the concerns raised, we have significantly expanded the limitations and future directions sections in the discussion section of the revised manuscript to address these critical points in greater depth.

Lastly, a minor typographical and grammatical revision is necessary throughout the manuscript to maintain professional standards, such as correcting the spelling of "Conrtol" to "Control" in Figure 2. A professional language review is recommended prior to resubmission.

Thank you for your careful review and valuable feedback. We appreciate your positive comments on the clarity of our results and the statistical analyses. Regarding the typographical error in Figure , we acknowledge the mistake and confirm that "Conrtol" will be corrected to "Control" in the revised version of the manuscript. We will thoroughly proofread the entire document to ensure no similar errors remain. Thank you again for your attention to detail and constructive suggestions.

In conclusion, the manuscript presents valuable and promising insights but requires substantial methodological clarifications, deeper acknowledgment of limitations, and a more comprehensive discussion before consideration for acceptance.

We sincerely appreciate the reviewer’s constructive feedback. In response to the concerns raised, we have significantly expanded the limitations and future directions sections in the discussion section of the revised manuscrip.

**Reviewer #3:**

The authors showed that a long non-coding RNA OIP5-AS1 is upregulated upon ischemia/reperfusion (I/R)-induced apoptosis, and can act like a sponge for miR-145-5p and release the miR-145-5p target (ROCK1) expression. Both in vitro (culture cells) and in vivo models were used.
I could read until Figure 2 end, but started to fell unconfortable for reading the result section afterwards. This is because Figures are not cited correctly in the Result section of maintext and the writing is poor especially in the latter half.

[Methods section]
“miR-145 mimics and inhibitors” information was not shown.

Thank you for pointing this out. We apologize for the oversight. The specific details regarding the miR-145 mimics and inhibitors have now been added to the revised manuscript.

[Result section]
In the paragraph “MiR-145-5p directly targeted by OIP5-AS1 and expressed at low levels in I/R”, Figure 3B is first cited, 3A cannot be found. Neither to 3C 3D 3F.
“Furthermore, silencing of OIP5-AS1 in I/R significantly upregulated miR-145-5p
expression (Fig. 2E).” – Figure 2E is not showing this evidence.
“These results indicated that miR-145-5p was downregulated in
I/R and was a target of OIP5-AS1.” – Better rephrase. Generally, a transcript is a target of miRNA.
“Western blot analysis
and RT-qPCR were performed to investigate ROCK1 expression. Results showed significant upregulation of ROCK1 in I/R cell models compared with controls (Fig.1F).” – Fig.1F is RNA data. No WB data was shown.
We sincerely thank the reviewer for pointing out the importance of ensuring consistency between figure citations and the main text descriptions. We have carefully reviewed all figure citations throughout the manuscript and we have verified that all figures are cited in the correct order and context within the text.We have revised the text to ensure that the descriptions of each figure panel are accurate and complete.

“The luciferase report assay demonstrated that miR-145-5p mimics substantially attenuated the luciferase activity of ROCK1-WT, while ROCK1-MUT showed no changes in response to miR-145-5p elevation (Fig. 3F).” – Need rephrase. More correctly, "no change after addition of miR-145-5p mimics.” It is very confusing that this figure is placed as Fig 3F.

The original sentence has been revised to: “The luciferase report assay demonstrated that miR-145-5p mimics significantly reduced the luciferase activity of ROCK1-WT, whereas no change was observed in ROCK1-MUT after the addition of miR-145-5p mimics.”

“ROCK1 expression was reduced in I/R by OIP5-AS1 deficiency while miR-145-5p was highly
expressed (Fig. 2F and 2G).” – These data are showing ROCK1 RNA and protein levels. but not miR-145-5p level.
“Altogether, OIP5-AS1 behaved as a ceRNA against miR-145-5p, resulting in ROCK1 upregulation.” – Cannot give such strong conclusion from these data. Just “suggest”.
Thank you for your careful reading and constructive feedback. We agree that the data presentation and conclusions need to be more precise.We have further verified and confirmed that all figure/table citations are consistent with their descriptions in the main text. And we carefully revised the statement in question to better reflect the preliminary nature of our findings: “Collectively, these data suggest OIP5-AS1 may behave as a ceRNA against miR-145-5p, resulting in ROCK1 upregulation.”

“ceRNA” appears here for the first time without explanation. What is it.
“Figure 5” appears in the last paragraph, without any explanation of individual panels (5A~E). Very poor and unfriendly writing.
We sincerely appreciate the reviewer's valuable feedback regarding the clarity of our manuscript. We have added a clear definition when first introducing the term:“ceRNA” . We have completely rewritten the paragraph to provide a comprehensive explanation of Figure 9.

[Discussion section]
“OIP5-AS1. OIP5-AS1 was found to inhibit the protective function of miR-145-5p, and it was confirmed to target miR-145-5p.” – “confirmed” sounds too strong compared to the presented data.

We sincerely appreciate the reviewer's valuable feedback regarding the clarity of our manuscript. We have rewritten the discussion section. For example, we have carefully revised the statement in question to better reflect the preliminary nature of our findings: Our findings indicate that OIP5-AS1 negatively regulates the cardioprotective effects of miR-145-5p through direct interaction, as demonstrated by our mechanistic studies.

The authors can try OIP5-AS1 overexpression experiment in the absence of IR treatment in vitro and in vivo. “a transcript is targeting a miRNA” is confusing, and better be rephrased, like “ OIP-AS1 can sequester miR-145-5p” or “OIP-AS1 has the target site of miR-145-5p”
Thank you for your careful reading and constructive feedback. We have rewritten the discussion section.

“This further supports the relationship between OIP5-AS1and miR-145-5p.” – should explain more about the “relationship” in this sentence.

Thank you for your careful reading and constructive feedback. We have rewritten the discussion section. We have carefully revised the statement: This finding further supports the competitive binding relationship between OIP5-AS1 and miR-145-5p, where OIP5-AS1 acts as a molecular sponge to miR-145-5p.
