# Supplementary material for: Downregulation of OIP5-AS1 inhibits apoptosis in myocardial ischemia/reperfusion injury via modulating the MiR-145-5p/ROCK1 axis
Source: PLoS One. 2025 May 20;20(5):e0324909. doi: 10.1371/journal.pone.0324909 (PMC12091815; doi:10.1371/journal.pone.0324909)
Supplement: Attachment — Submitted filename: Response_to_Reviewers_auresp_2.docx [file pone.0324909.s006.docx]

Dear Editor:

Thank you very much for your letter and advice about our manuscript with the mumber of PONE-D-25-10451. According with reviewer’s comments, we revised the relevant parts, and these parts were highlighted in the manuscript. All of the questions were answered below.

**Journal Requirements:**

Please review your reference list to ensure that it is complete and correct. If you have cited papers that have been retracted, please include the rationale for doing so in the manuscript text, or remove these references and replace them with relevant current references. Any changes to the reference list should be mentioned in the rebuttal letter that accompanies your revised manuscript. If you need to cite a retracted article, indicate the article’s retracted status in the References list and also include a citation and full reference for the retraction notice.

We have carefully reviewed our reference list to ensure its accuracy and completeness. We screened all cited references using PubMed to confirm that none of the cited articles have been retracted. All references are current and valid.

Reviewer #1: Thank you for conducting all the experiments I suggested and for including the corresponding results.

We sincerely appreciate your insightful comments and are grateful for your acknowledgment of our work in implementing all suggested experiments. Your expertise has significantly strengthened our study.

Reviewer #2: I have carefully evaluated the revised manuscript entitled "Downregulation of OIP5-AS1 Inhibits Apoptosis in Myocardial Ischemia/Reperfusion Injury via Modulating the MiR-145-5p/ROCK1 Axis."

I appreciate the authors' efforts to thoroughly address all the concerns raised in the initial review. The manuscript has undergone significant improvements, particularly in the following aspects:

Methodology: The authors have now provided detailed descriptions of the experimental procedures, including the concentrations of siRNAs, mimics, and inhibitors, the rationale for choosing Lipofectamine 2000, and optimization details. The methodology is now sufficiently transparent to allow reproducibility.

Controls: Appropriate negative controls were included and clearly described, addressing concerns about potential nonspecific effects of transfection reagents.

Results Presentation: Minor typographical errors, such as the correction of "Conrtol" to "Control," have been rectified.

Discussion and Limitations: The authors have expanded the discussion significantly, explicitly acknowledging key limitations and proposing valuable future research directions, which strengthen the manuscript's scientific rigor and translational relevance.

Language and Style: The manuscript's language has been improved considerably. Although minor editorial polishing could further enhance clarity, it does not hinder comprehension or scientific value.

Given the authors' careful and thoughtful revisions, I find that all my initial concerns have been satisfactorily addressed.

Therefore, I recommend the manuscript for acceptance after minor language editing (optional proofreading).

Congratulations to the authors for their excellent work and for the significant improvements made to the manuscript.

We are deeply grateful for your insightful comments that significantly improved our manuscript. We have implemented the minor language edits as suggested.

Reviewer #3: Many points were addressed by the authors.

I just give some points that would need correction before publication.

Table 1

si-OIP5-AS1 and miR mimic‑NC contain "T" in their sequence. If true, why control does not have them.

"Concentration" cannot be understood. Final concentration in culture?

We sincerely appreciate the reviewer's meticulous review. SiRNA sequences are shown as DNA templates (containing Thymine). MiRNA mimics/inhibitors are direct synthetic RNA oligos (containing Uracil). We have modified the si-NC sequence to be consistent with DNA template notation (using Thymine instead of Uracil).

We apologize for the ambiguity. The concentrations listed are indeed the final working concentrations in cell culture medium after transfection complex delivery. We have revised the table header to ‘Final working concentration (nM)’.

Table 2

Oligos for miRNA would not be correct. Probably the reverse one is an adaptor for RT. Universal Rv primer should be used here.

I have no idea if "miR, microRNA; ROCK1, Rho-associated coiled-coil-containing kinase 1" included in Table 2 is needed in this position.

We sincerely appreciate the reviewer's expert insights regarding Table 2. We have removed the redundant reverse primer sequence and moved "miR, microRNA; ROCK1..." to the general abbreviation list in the Table 2 footer.

Line 246

Sentence stopped in the middle. "compared" to what.

We sincerely appreciate the reviewer's careful reading. Regarding the incomplete sentence at Line 246 in the original manuscript, we have revised the full sentence “Compared with the control group, expression of miR-145-5p in H9c2 cardiomyocytes was significantly downregulated in I/R.”

Line 268

I didn't get what "pivoral" indicates here.

We sincerely appreciate the reviewer's careful reading and constructive feedback. We have modified the text to “The results suggested that ROCK1 played a significant role in mediating OIP5-AS1's effects on cardiomyocyte apoptosis in I/R.”

Line 275

Figure 5 and "8"

8A and 8B are not cited, although authors stated all Figures are to be correctly cited.

We sincerely appreciate the reviewer's careful reading and constructive suggestions. We have now ensured all figure panels are properly cited throughout the text. The relevant paragraph now reads “ In the present study, we initially corroborated the efficacy of OIP5-AS1 knockdown in our experimental models (Fig. 5A and 8A). Importantly, OIP5-AS1 deficiency led to a substantial decrease in ROCK1 expression, accompanied by an increase in miR-145-5p levels under I/R conditions (Fig. 5B-C and 8B-C). ”(Line 282)

Figure legends for Fig1~9 were provided as supporting information.

We sincerely appreciate the reviewer's careful reading. We have now uploaded all figure legends (for Fig1-9) as Supporting Information S4 File.
